# Supplementary material for: Distinct isoforms of Nrf1 diversely regulate different subsets of its cognate target genes
Source: Sci Rep. 2019 Feb 27;9:2960. doi: 10.1038/s41598-019-39536-0 (PMC6393581; doi:10.1038/s41598-019-39536-0)
Supplement: Supplementary file 1 — Supplementary Info File [file 41598_2019_39536_MOESM1_ESM.pdf]

Distinct isoforms of Nrf1 diversely regulate different subsets of its cognate target genes

Meng Wang, Lu Qiu, Xufang Ru, Yijiang Song, Yiguo Zhang*

The Laboratory of Cell Biochemistry and Topogenetic Regulation, College of Bioengineering and Faculty of Sciences, Chongqing University, No. 174 Shazheng Street, Shapingba District, Chongqing 400044, China

*Correspondence should be addressed to Yiguo Zhang (email: yiguozhang@cqu.edu.cn or eaglezhang64@gmail.com).

Supplementary information:

Supplementary table S1: Alignment statistics of the primary sequencing data.
Supplementary table S2: The differentially expressed genes regulated by Nrf1α.
Supplementary table S3: The differentially expressed genes regulated by Nrf1β.
Supplementary table S4: The differentially expressed genes regulated by Nrf1γ.
Supplementary table S5: Gene ontology enrichment analysis of DEGs regulated by Nrf1α.
Supplementary table S6: Gene ontology enrichment analysis of DEGs regulated by Nrf1β.
Supplementary table S7: Gene ontology enrichment analysis of DEGs regulated by Nrf1γ.
Supplementary table S8: KEGG pathway enrichment analysis of DEGs regulated by Nrf1α.
Supplementary table S9: KEGG pathway enrichment analysis of DEGs regulated by Nrf1β.
Supplementary table S10: KEGG pathway enrichment analysis of DEGs regulated by Nrf1γ.
Supplementary table S11: The top ranked DEGs in heatmap.
Supplementary table S12: The top ranked DEGs in gene-regulatory network.
Supplementary table S13: Gene expression in network of Nrf1 from BioGRID database.
Supplementary table S14: Gene expression in network of Nrf1 from STRING database.
Supplementary table S15: Expression of bZIP factors regulated by Nrf1 isoforms.
Supplementary table S16: Expression of proteasomes regulated by Nrf1 isoforms.
Supplementary table S17: Distinct paired primers used for expression constructs and RT-qPCR analysis.

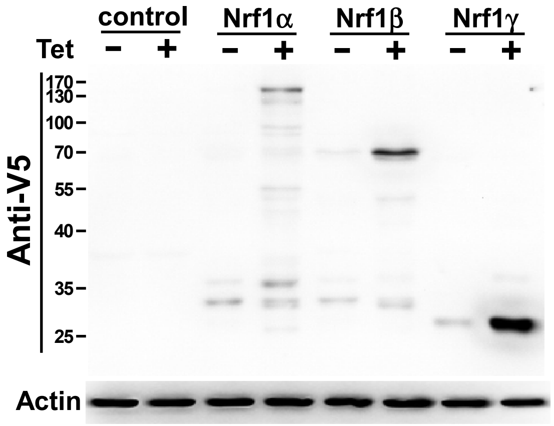

low-contrast of blot in figure 1 C

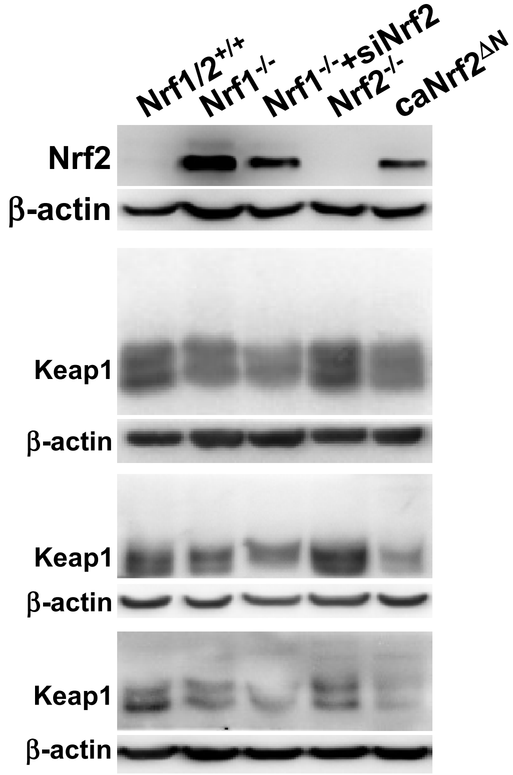

low-contrast of blots in figure 4 D

Table S17. Distinct paired primers used for expression constructs and RT-qPCR analysis

Name	Forward (5'-3')	Reverse (5'-3')
1, The following primers used for expression plasmids		
Nrf1α	CGGGGTACCATGCTTTCTCTGAAG AAATACTTAACGGAAG	CCGGATATCCCTTTCTCCGGTCC TTTGGCTTCCTCTCCTG
Nrf1β	CGGGGTACCATGGAAGTGAACACA TCAGCAAGTGAAATC	
Nrf1γ	CGGGGTACCATGGCACCCAGTGCC CTGGACTCAGCCGAC	
2, The following primers used for RT-qPCR analysis		
Nrf1	GGAGGAGTTCAATGAACTGCTGTC	CTCTGGACCTTCTGCTTCATCTGT
KEAP1	AACAACTCGCCCGACGGCAACAC	CATCCCGCTCTGGCTCATACCTC
PSMA1	GGCTTACTGCTGATGCTAGACTG	GAGATACAAGACGAGACACAGGC
PSMA4	GTCCCTTTGGTGTTTCATTGCTG	CTTCCATCCCCCGTAATTTCCAC
PSMB7	CTGGATTTTCTCCGCCCATACAC	GTTTGGACTGTTTCTTCCAGCAC
PSMC2	TACAGGTTGCCAGGTGTACAAAG	ACCTGATCACTAAGGTCCACCAC
PSMC6	AGGTCCCATTACAAAGCATGGTG	AGCACGAATTGCGAACATACCTG
PSMD12	TATCTGTTGATGAGTCCGAAGCC	GATCCTTGGGTCTCTGGAAGTTG
HO1	CAGAGCCTGGAAGACACCCTAA	AAACCACCCCAACCCTGCTAT
GCLM	GTGTGATGCCACCAGATTTGAC	CACAATGACCGAATACCGCAGT
GCLC	ACTCTGCCTATGTGGTGTTTGTG	CATTCCCTGCAAGACAGCATCTC
MT1E	ATGGACCCCAACTGCTCTTGCGCCA	ACAGCAGCTGCACTTCTCCGATG
PGC1β	AAAATCTCTCCAGCGACATGAGC	CGCCTCTCCTATTTCTTGTCAGC
LPIN1	CCTAAAGGAGAGCTGGTACAGGA	GAATGGCTTCTTTTCAGCAACGG
NPM1	TTCAGGGCCAGTGCATATTAGTG	GGCAGACCGCTTTCCAGATATAC
ESD	GGCTTATGATGCTACCCACCTTG	GGCAGCTATGAAGTTATCAGGGAG
IPO5	AATTGCGGAAGGAGAAATGCAC	CCAGAAGTCTGTACTTGGCGAAC
IFITM1	GTGATCAACATCCACAGCGAGAC	TCTTCCTGTCCCTAGACTTCACG
HSPD1	AACTTTCAGATGGAGTGGCTGTG	AGCAGCTCTTGTAGCATTAAGGG
KPNB1	AGGGATTAAAGGGGGATCAGGAG	CCTATTAGTCCAGCAGCACAAGC
FOXC1	ATAGCTACATCGCGCTCATCAC	CTGCTTGTTGTCCCGGTAGAAG
ELOVL5	TACAACTTCTTCTGTCAGGGCAC	AAGTAGTACCACCAGAGGACACG
ID3	GACGACATGAACCACTGCTACTC	GGATTTCCACCTGGCTAAGCTG
KRT19	GGACTGAAGAATTGAACCGGGAG	GACTGCAGCTCAATCTCAAGACC
TRAPPC2L	TGGTAGATTCCTCCAACACAGC	TGTAGAAGGGGTTGCACATCAC
β-actin	CATGTACGTTGCTATCCAGGC	CTCCTTAATGTCACGCACGAT
