# Supplementary material for: Distinct isoforms of Nrf1 diversely regulate different subsets of its cognate target genes
Source: Sci Rep. 2019 Feb 27;9:2960. doi: 10.1038/s41598-019-39536-0 (PMC6393581; doi:10.1038/s41598-019-39536-0)
Supplement: Supplementary file 2 — Dataset 1 [file 41598_2019_39536_MOESM2_ESM.zip › supplementary-TableS17-Primers.pdf]

Table S17. Distinct paired primers used for expression constructs and RT-qPCR analysis

Name	Forward (5'-3')	Reverse (5'-3')
**1, The following primers used for expression plasmids**		
Nrf1α	CGGGGTACCATGCTTTCTCTGAAG AAATACTTAACGGAAG	CCGGATATCCCTTTCTCCGGTCC TTTGGCTTCCTCTCCTG
Nrf1β	CGGGGTACCATGGAAGTGAACACA TCAGCAAGTGAAATC	
Nrf1γ	CGGGGTACCATGGCACCCAGTGCC CTGGACTCAGCCGAC	
**2, The following primers used for RT-qPCR analysis**		
Nrf1	GGAGGAGTTCAATGAACTGCTGTC	CTCTGGACCTTCTGCTTCATCTGT
KEAP1	AACAACTCGCCCGACGGCAACAC	CATCCCGCTCTGGCTCATACCTC
PSMA1	GGCTTACTGCTGATGCTAGACTG	GAGATACAAGACGAGACACAGGC
PSMA4	GTCCCTTTGGTGTTTCATTGCTG	CTTCCATCCCCCGTAATTTCCAC
PSMB7	CTGGATTTTCTCCGCCCATACAC	GTTTGGACTGTTTCTTCCAGCAC
PSMC2	TACAGGTTGCCAGGTGTACAAAG	ACCTGATCACTAAGGTCCACCAC
PSMC6	AGGTCCCATTACAAAGCATGGTG	AGCACGAATTGCGAACATACCTG
PSMD12	TATCTGTTGATGAGTCCGAAGCC	GATCCTTGGGTCTCTGGAAGTTG
HO1	CAGAGCCTGGAAGACACCCTAA	AAACCACCCCAACCCTGCTAT
GCLM	GTGTGATGCCACCAGATTTGAC	CACAATGACCGAATACCGCAGT
GCLC	ACTCTGCCTATGTGGTGTTTGTG	CATTCCCTGCAAGACAGCATCTC
MT1E	ATGGACCCCAACTGCTCTTGCGCCA	ACAGCAGCTGCACTTCTCCGATG
PGC1β	AAAATCTCTCCAGCGACATGAGC	CGCCTCTCCTATTTCTTGTCAGC
LPIN1	CCTAAAGGAGAGCTGGTACAGGA	GAATGGCTTCTTTTCAGCAACGG
NPM1	TTCAGGGCCAGTGCATATTAGTG	GGCAGACCGCTTTCCAGATATAC
ESD	GGCTTATGATGCTACCCACCTTG	GGCAGCTATGAAGTTATCAGGGAG
IPO5	AATTGCGGAAGGAGAAATGCAC	CCAGAAGTCTGTACTTGGCGAAC
IFITM1	GTGATCAACATCCACAGCGAGAC	TCTTCCTGTCCCTAGACTTCACG
HSPD1	AACTTTCAGATGGAGTGGCTGTG	AGCAGCTCTTGTAGCATTAAGGG
KPNB1	AGGGATTAAAGGGGGATCAGGAG	CCTATTAGTCCAGCAGCACAAGC
FOXC1	ATAGCTACATCGCGCTCATCAC	CTGCTTGTTGTCCCGGTAGAAG
ELOVL5	TACAACTTCTTCTGTCAGGGCAC	AAGTAGTACCACCAGAGGACACG
ID3	GACGACATGAACCACTGCTACTC	GGATTTCCACCTGGCTAAGCTG
KRT19	GGACTGAAGAATTGAACCGGGAG	GACTGCAGCTCAATCTCAAGACC
TRAPPC2L	TGGTAGATTCCTCCAACACAGC	TGTAGAAGGGGTTGCACATCAC
β-actin	CATGTACGTTGCTATCCAGGC	CTCCTTAATGTCACGCACGAT
