# Supplementary material for: Distinct isoforms of Nrf1 diversely regulate different subsets of its cognate target genes
Source: Sci Rep. 2019 Feb 27;9:2960. doi: 10.1038/s41598-019-39536-0 (PMC6393581; doi:10.1038/s41598-019-39536-0)
Supplement: Supplementary file 2 — Dataset 1 [file 41598_2019_39536_MOESM2_ESM.zip › supplementary-TableS15-bZIP factors.doc]

| **Gene Symbol** | **Description** | **RPKM** | | | | **Log_2_ (fold change)** | | |
| --- | --- | --- | --- | --- | --- | --- | --- | --- |
|  |  | **control** | $\alpha$ | $\beta$ | $\gamma$ | $\alpha$**/control** | $\beta$**/control** | $\gamma$**/control** |
| NFE2L1 | nuclear factor (erythroid-  derived 2)-like 1 | 69.25 | 1675.62 | 183.49 | 784.67 | 4.60 | 1.41 | 3.50 |
| NFE2L2 | nuclear factor (erythroid-  derived 2)-like 2 | 4.81 | 9.87 | 12.86 | 5.42 | 1.04 | 1.42 | 0.17 |
| NFE2L3 | nuclear factor (erythroid-  derived 2)-like 3 | 2.62 | 3.20 | 3.63 | 3.47 | 0.29 | 0.47 | 0.41 |
| BACH1 | BTB and CNC homology 1, basic leucine zipper transcription factor 1 | 0.96 | 1.63 | 1.63 | 0.68 | 0.77 | 0.77 | -0.49 |
| BACH2 | BTB and CNC homology 1, basic leucine zipper transcription factor 2 | 1.90 | 2.38 | 2.98 | 2.07 | 0.33 | 0.65 | 0.12 |
| MAF | v-maf musculoaponeurotic fibrosarcoma oncogene  homolog (avian) | 3.21 | 2.88 | 8.40 | 2.82 | -0.16 | 1.39 | -0.18 |
| MAFA | v-maf musculoaponeurotic fibrosarcoma oncogene  homolog A (avian) | 0.91 | 1.48 | 2.67 | 1.61 | 0.70 | 1.55 | 0.82 |
| MAFB | v-maf musculoaponeurotic fibrosarcoma oncogene  homolog B (avian) | 2.53 | 2.58 | 5.19 | 4.76 | 0.03 | 1.04 | 0.91 |
| MAFF | v-maf musculoaponeurotic fibrosarcoma oncogene  homolog F (avian) | 1.62 | 1.14 | 0.96 | 1.59 | -0.50 | -0.75 | -0.03 |
| MAFG | v-maf musculoaponeurotic fibrosarcoma oncogene  homolog G (avian) | 12.01 | 12.90 | 12.16 | 11.96 | 0.10 | 0.02 | -0.01 |
| MAFK | v-maf musculoaponeurotic fibrosarcoma oncogene  homolog K (avian) | 5.39 | 6.22 | 3.63 | 6.10 | 0.21 | -0.57 | 0.18 |
